# Pre-symbiotic response of the compatible host spruce and low-compatibility host pine to the ectomycorrhizal fungus *Tricholoma vaccinum*

**DOI:** 10.3389/fmicb.2023.1280485

**Published:** 2023-12-04

**Authors:** Marycolette Ndidi Ezediokpu, Rayko Halitschke, Katrin Krause, Wilhelm Boland, Erika Kothe

**Affiliations:** ^1^Microbial Communication, Institute of Microbiology, Friedrich Schiller University, Jena, Germany; ^2^Bioorganic Chemistry, Max Planck Institute for Chemical Ecology, Jena, Germany; ^3^Mass Spectrometry and Metabolomics, Max Planck Institute for Chemical Ecology, Jena, Germany

**Keywords:** ectomycorrhiza, Tricholoma vaccinum, fungal volatilome, phytohormones, host tree metabolome, *Picea abies*, *Pinus sylvestris*

## Abstract

Mutualistic ectomycorrhizal symbiosis requires the exchange of signals even before direct contact of the partners. Volatiles, and specifically volatile terpenoids, can be detected at a distance and may trigger downstream signaling and reprogramming of metabolic responses. The late-stage ectomycorrhizal fungus *Tricholoma vaccinum* shows high host specificity with its main host spruce, *Picea abies,* while rarely associations can be found with pine, *Pinus sylvestris*. Hence, a comparison of the host and the low-compatibility host’s responses can untangle differences in early signaling during mycorrhiza formation. We investigated sesquiterpenes and identified different patterns of phytohormone responses with spruce and pine. To test the specific role of volatiles, trees were exposed to the complete volatilome of the fungus versus volatiles present when terpene synthases were inhibited by rosuvastatin. The pleiotropic response in spruce included three non-identified products, a pyridine derivative as well as two diterpenes. In pine, other terpenoids responded to the fungal signal. Using exposure to the fungal volatilome with or without terpene synthesis inhibited, we could find a molecular explanation for the longer time needed to establish the low-compatibility interaction.

## 1 Introduction

Interactions between fungi and plants span the continuum between pathogenic, commensal to mutually beneficial symbiotic interactions (Abdulsalam et al., 2019). This is also true for the mutually beneficial ectomycorrhizal symbiosis between trees and basidiomycete fungi like the edible russet scaly tricholoma, *Tricholoma vaccinum*. The fungus is a slow growing, late-stage succession mycorrhizal fungus forming long exploration type morphotypes on the short roots, which allow for wide range exploration and sustainable delivery of nutrients and water to the tree (Agerer, 2001). The soil mycelium connects host trees all along the growth path, adding to ecosystem stability becoming ever more important with climate change (Peter, 2006). The late-stage mycorrhiza is expected to mount a slow, but sustainable reaction in both partners, while for early-stage mycorrhiza a fast reaction to override plant defense responses would be required to allow for symbiosis (Raudaskoski and Kothe, 2015).

In ectomycorrhizal associations, *T. vaccinum* is mainly observed with fruiting bodies occurring under spruce and only rarely found under pine; although both mycorrhizae have been successfully re-created in the laboratory (Wagner et al., 2015; Sammer et al., 2016). While the interaction with the compatible host *Picea abies* is faster and more complete, the interaction with *P. sylvestris* needs longer for establishment, and the Hartig’ net is not fully developed even after longer periods (Sammer et al., 2016). The specificity in host interaction allows to dissect early responses in compatible versus low-compatibility interactions to gain insight into signals priming plant defense that have not been comprehensively studied with regard to compatibility of the ectomycorrhizal partner organisms.

Stable symbiotic associations need to overcome and disarm plant defenses, which requires specific communication between both partners, likely even before they come into physical contact (Almario et al., 2022). The fully established mutually beneficial symbiosis then improves the tree’s nutrient acquisition from the soil, while the fungus is supplied by the tree with carbohydrates from photosynthesis. Plant responses are best known from plant pathogen interactions, including trans-kingdom communication at a distance. There, pattern recognition receptors allow to specifically respond to pathogen/microbe-associated patterns (PAMPs/MAMPs) that induce chemical defenses by production of, e.g., terpenoids, phenolics, or alkaloids (Pfabel et al., 2012). A hypersensitive response with formation of reactive oxygen species is also mounted and, finally, in incompatible plant pathogenic interactions, programmed cell death leads to concomitant killing especially of the biotrophic invading fungi. Since a mutual symbiosis needs to allow for plant invasion by the mycorrhizal fungus, this general response needs to be minimized. Hence, early signals need to be recognized to allow for plant priming and establishing a fruitful and co-operative symbiosis (Abdulsalam et al., 2019; Wong et al., 2019).

In addition to the direct response mounted upon receiving a PAMP signal, a long-lasting response can be induced. Systemic acquired resistance (SAR) involves salicylic acid signaling, leading to accumulation of pathogenesis-related proteins via regulation of transcription factors (for review: Durrant and Dong, 2004; Pieterse et al., 2014). Induced systemic resistance (ISR) leads to increased formation of a spectrum of antimicrobial compounds including plant phytoalexins (Ahuja et al., 2012; Pieterse et al., 2014). Another compound, 12-oxo-phytodienoic acid (OPDA), has been identified as an intermediate in jasmonate biosynthesis, but also to act independently as a signal for different stressors including the responses to pathogen-related damage as well as osmotic stress or wounding (Koch et al., 1999; Kessler and Baldwin, 2002). Mycorrhizae can relieve such stress (Shi et al., 2023).

Here, we use the highly host-specific interaction of *T. vaccinum* with *P. abies* and the low-compatibility interaction with *P. sylvestris* to identify the characteristic responses of the host trees to volatiles emitted by the fungus. The fungal volatilome was reduced for terpenoids by adding a terpene synthase inhibitor, rosuvastatin. The trees’ phytohormone and metabolome responses to the early signals transmitted through volatiles were scored. In our earlier studies, we had identified at least 20 terpenoids formed by *T. vaccinum* enriched in interactions with the tree host *P. abies*, and identified nine terpene synthase genes (Abdulsalam et al., 2019, 2022; Ezediokpu et al., 2022). Here, we wanted to identify specific metabolic reactions in both compatible and low-compatibility hosts associated with the recognition of terpenoid signals produced by the fungus prior to mycorrhiza formation.

## 2 Materials and methods

### 2.1 Cultivation

*Tricholoma vaccinum* GK6514 (SF004731, Jena Microbial Resource Collection, Jena, Germany) was cultivated on agar modified Melin Nokrans medium (MMNb; Kottke et al., 1987) at 23°C for 4 weeks*. Picea abies* and *Pinus sylvestris* seeds (Landesforst Mecklenburg-Vorpommern, Germany) were immersed in tap water overnight, surface-sterilized using 30% H_2_O_2_ for 90 min, washed several times with sterile distilled water to eliminate traces of H_2_O_2_, and germinated on a germination agar for 2–4 weeks (Chilvers et al., 1986).

For co-cultivation without direct contact, but enabling volatile exchange, a nested set-up was created. In a large Petri dish of 12 cm diameter filled with MMNa agar (MMNb with 0.5 g/L (NH_4_)_2_HPO_4_ without malt extract; Kottke et al., 1987) and containing the tree partner, a smaller Petri dish was placed that contained MMNb and was inoculated with *T. vaccinum* (n ≥ 4). The set-up was incubated in a climate chamber with a daylight cycle of 12 h at 23/17°C at 80% humidity. To evaluate the importance of sesquiterpenes in the volatile blend exchanged, rosuvastatin (calcium salt, 5–7 ng/mL) was added to the agar for *T. vaccinum*. The addition of rosuvastatin had no obvious effect on the fungal growth. To allow direct contact for control experiments, *P. abies* with or without *T. vaccinum* was grown on MMNa. After 5–6 weeks, the trees were harvested by flash freezing in liquid nitrogen and stored at −80°C.

### 2.2 Phytohormone identification and quantification

Flash-frozen seedlings were ground in liquid nitrogen using a mortar and pestle and subsequently transferred into reaction tubes containing two steel balls and stored at 20°C for 30 min, before being homogenized in the extraction buffer (80% MeOH) and incubated at 20°C overnight. Grinding and maceration was performed in a tissue homogenizer (1,600 MiniG, SPEX SamplePrep; Metuchen, NJ) for 60 s at 1150 strokes min^−1^. After centrifugation for 20 min at 4°C, the supernatant was collected and phytohormones were analyzed by triple-quadrupole MS using isotope-labeled internal standards in the extraction buffer (Schäfer et al., 2016).

The phytohormones abscisic acid (ABA), salicylic acid (SA), 12-oxo-phytodienoic acid (OPDA) jasmonic acid (JA) and its amino acid derivatives JA-Ile, JA-Met, JA-Phe, JA-Trp, JA-Val, as well as hydroxy-jasmonic acid, hydroxy-and carboxy-jasmonoyl-isoleucine were quantified using internal standards: 10 ng D_6_-jasmonic acid, 10 ng D_4_-abscisic acid, 10 ng D_6_-salicylic acid, and 10 ng of D_6_-JA-Ile. Supernatants were analyzed by liquid chromatography/mass spectrometry on an EvoQ-Elite-QQQ-MS (Bruker) connected to an UltiMate 3000RS UHPLC system (Thermo Fisher, Waltham). The LC was equipped with a Zorbax Eclipse XDB-C18 column (3.0 × 50 mm, 1.8 m; Agilent, Santa Clara) and mobile phases A (0.05% formic acid and 0.1% acetonitrile in water) and B (MeOH) were applied in a gradient at a flow rate of 400 μL min^−1^ [time (min) / % mobile phase B: 0/5, 0.5/5, 0.6/50, 2.5/100, 3.5/100, 3.55/5, 4.5/5]. The sample injection volume was 2 L. ESI parameters in negative ionization mode were: 4500 V ion spray voltage, 350°C cone temperature, 20 psi cone gas flow, 400°C heated probe temperature, 50 psi nebulizer gas flow, and 1.5 mTorr collision gas were employed. Multiple reaction monitoring (MRM) settings for the detection of phytohormones and internal standards were recently described (Schäfer et al., 2016).

### 2.3 Metabolome analyses

To investigate changes in the metabolome in an untargeted approach, the MeOH extracts were analyzed by ultra-high performance liquid chromatography - QTOF MS. An UltiMate 3,000 rapid separation LC system (Thermo Fisher, Waltham) equipped with a Thermo Fisher Acclaim RSLC 120 C18 column (2.1 × 150 mm, 2.2 μm) was used with a 25-min linear gradient from 5–95% mobile phase B (mobile phase A: water with 0.05% formic acid and 0.1% acetonitrile; mobile phase B: acetonitrile containing 0.05% formic acid) and a flow rate of 0.3 mL min^−1^. Full scan MS data (m/z 100–1,500) was recorded on an impact II Q-TOF MS system (Bruker Daltonics, Berlin) with electrospray ionization (ESI) in positive ion mode: end plate offset of 500 V, capillary voltage 4,500 V, capillary exit 130 V, dry temperature 180°C, and a dry gas flow of 10 mL min^−1^. For mass calibration, sodium formate (250 mL isopropanol, 1 mL formic acid, 5 mL 1 M NaOH in 500 mL MilliQ water) was infused at the beginning of each sample run and data files were calibrated with the Bruker high-precision calibration algorithm. Instrument control, data acquisition, and reprocessing were performed using HyStar 3.1 (Bruker Daltonics, Berlin). In addition to the full scan MS data, data-dependent MS^2^ data were recorded using the Bruker autoMSMS function and mass features and corresponding MS^2^ spectra were extracted with the Metaboscape software (Bruker Daltonics, Berlin).

### 2.4 Data analysis and statistical treatments

Statistical analyses were performed for the Metaboscape-extracted mass features with metaboanalyst (www.metaboanalyst.ca; last access Dec 1st, 2022), which proceeded by data normalization by the mean, data transformation deployed log transformation while scaling was by Pareto scaling in R (search.r-project.org/CRAN/refmans/IMIFA/html/pareto_scale.html; last access Dec 1st, 2022), which exploits the mean centered divided by the square root of the standard deviation.

Multivariate analysis ANOVA and partial least square analysis were carried out using R. The metabolites with *p*-values <0.1 and fold change ≥2 were considered as differentially abundant metabolites. Structural proposals for individual metabolites were determined using Sirius (Dührkop et al., 2019), Canopus (Dührkop et al., 2015), CSI:finger ID (Hoffmann et al., 2021), CSI:finger Cosmic (Böcker and Dührkop, 2016), fragmentation tree (Böcker and Dührkop, 2016) and Zodiac (Ludwig et al., 2020).

## 3 Results

### 3.1 Terpene synthase inhibition leads to a reduction of the terpenoid spectrum in the fungal volatilome

**Figure 1:**
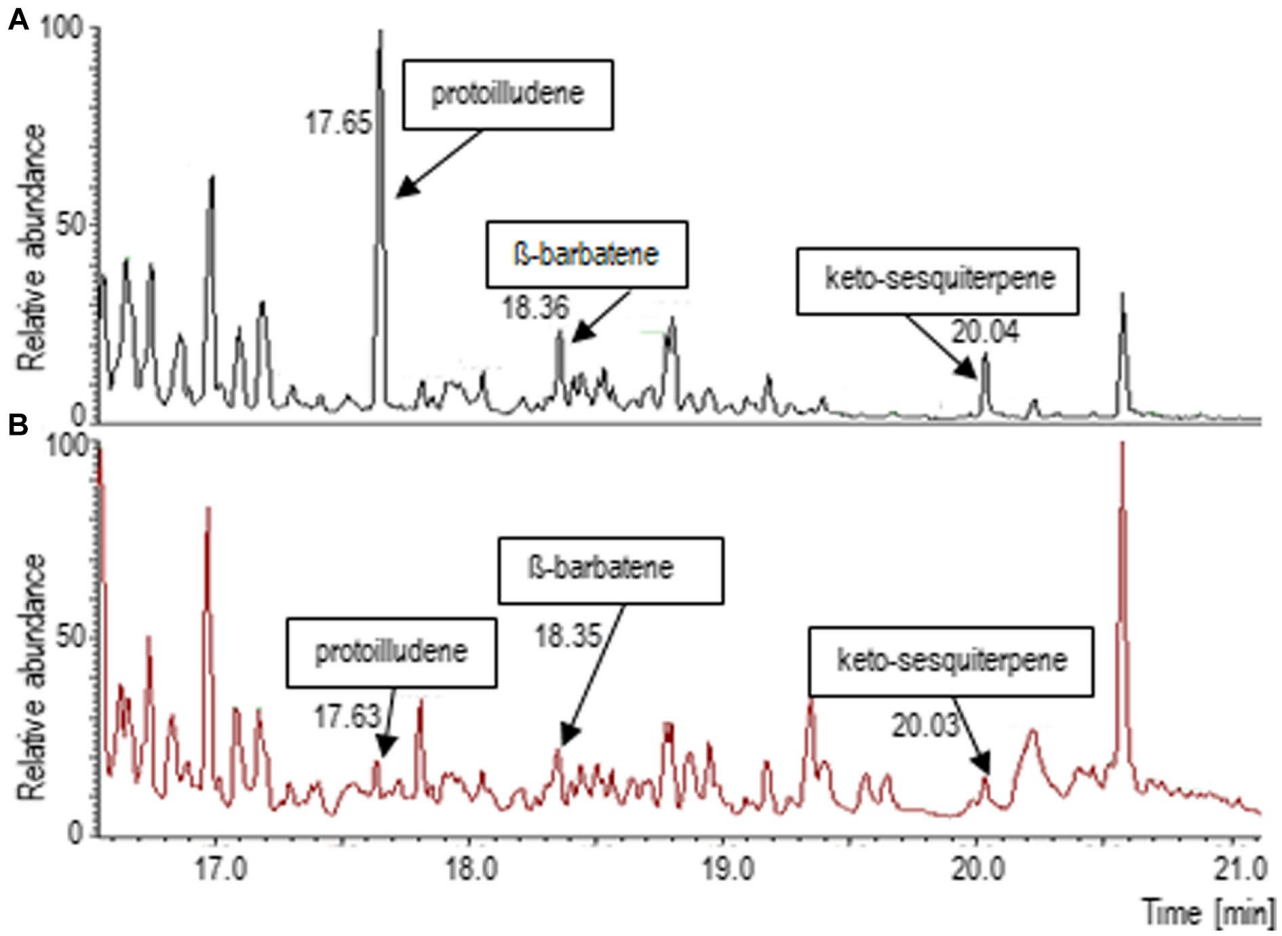
Terpenoid spectrum changes after application of the terpenoid synthesis inhibitor rosuvastatin. **(A)** Gas chromatogram of the head space of *Tricholoma vaccinum* growing with the full volatilome, **(B)** growth on media with rosuvastatin. Major terpenes of *Tricholoma vaccinum* were identified as Δ6-protoilludene (17.63 min), *β*-barbatene (18.36 min) and a keto-sesquiterpene (20.04 min; compare Ezediokpu et al., 2022).

Since a complex spectrum of terpenoids had been found in the headspace of *T. vaccinum* that was grown in symbiosis with the host *P. abies* (Ezediokpu et al., 2022), the terpene synthase inhibitor rosuvastatin was used to suppress terpene biosynthesis in the fungus. Consequently, the major mycorrhiza-regulated compounds, protoilludene and an unknown keto-metabolite, as well as -barbatene were found in much lower abundance (Figure 1). Thus, the reduced volatile spectrum of *T. vaccinum* could be used to compare the effect of terpenoids on the compatible and the low-compatibility host interaction.

**Figure 2:**
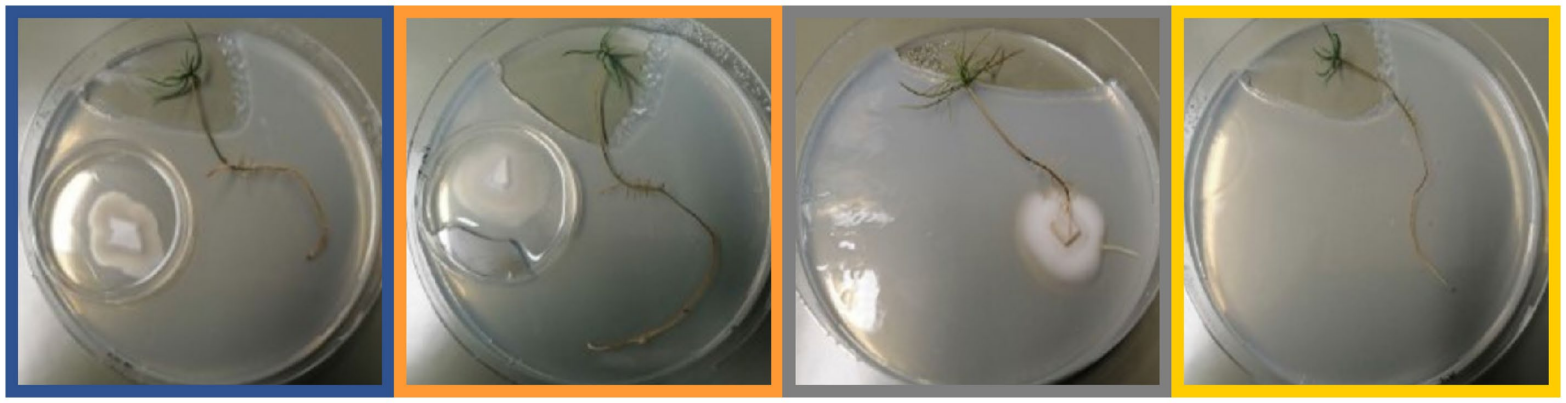
Experimental approach to sample tree seedling (approx. 9 weeks old) for phytohormone and metabolome analyses in treatments exposed to volatiles of *Tricholoma vaccinum* (blue frame around picture), to volatiles of *Tricholoma vaccinum* grown with the terpene synthase inhibitor rosuvastatin (orange frame), in direct contact (grey frame) or without the fungus (yellow frame).

In a new experimental system, two compartments were created by placing a smaller Petri dish into a larger. While the larger one was holding the tree seedling, the smaller one contained medium on which *T. vaccinum* was inoculated, with or without rosuvastatin (Figure 2). This enabled us to evaluate volatile exchange from the fungus with a complete set of volatiles and from the inhibited fungi with a reduced spectrum of volatiles. Controls with the tree alone were combined with a control, in which direct mycorrhizal contact was allowed. With this approach it became possible to compare the levels of phytohormones and metabolites in the respective tree exposed to the fungal volatilome with or without direct contact.

### 3.2 Phytohormone response to terpene signaling

**Figure 3:**
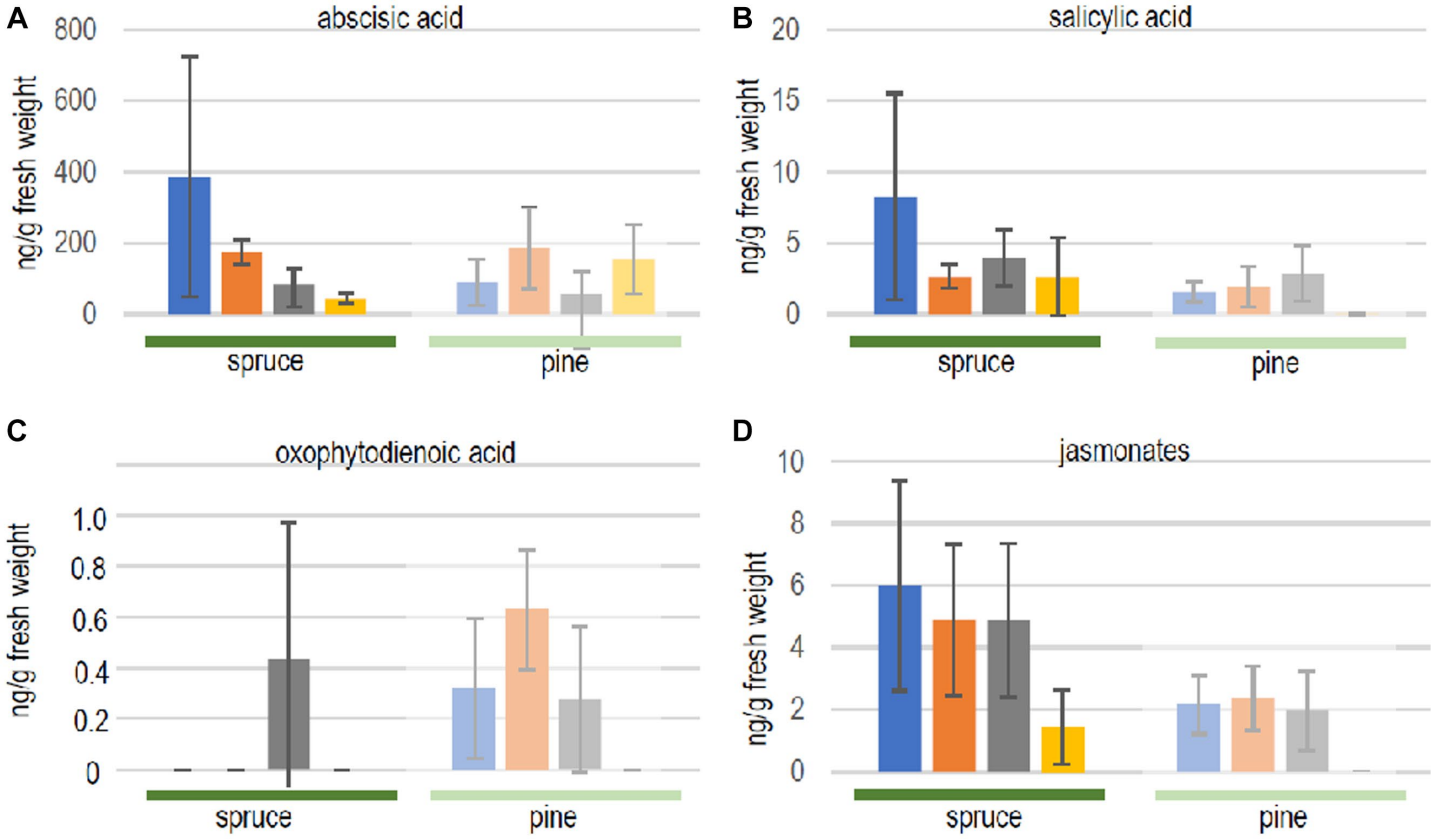
Phytohormone quantification in seedlings of spruce (full color, dark green line) and pine (dull color, light green line) after contact to full volatilome of *Tricholoma vaccinum* (blue), to terpene reduced volatilome (orange), in direct contact (grey) or without the fungus (yellow): **(A)** abscisic acid, **(B)** salicylic acid, **(C)** 12-oxophytodienoic acid OPDA, **(D)** jasmonates. For the sum of jasmonates, jasmonic acid and its −isoleucine, −methionine, −phenylalanine, −tryptophan, and-valine conjugates were added up (single values see Supplementary Table S1), *n* ≥ 4.

First, it was evaluated whether both, the compatible host spruce and the low-compatibility host pine, responded to volatiles of the ectomycorrhizal fungus differently. Phytohormone levels, indeed, showed a different response comparing both hosts (Figure 3). The exposure to fungal volatiles resulted in markedly different patterns between both trees. However, reduced terpene production in the fungus yielded no significant changes in seedling phytohormone responses compared to the exposure to the full volatilome.

In spruce trees, levels of abscisic acid were significantly elevated in response to the full and terpene-deficient reduced volatilome compared to control seedlings without fungus, indicating a response to early, volatile transmitted recognition (see Figure 3). In contrast, neither salicylic acid nor jasmonates were significantly changed (albeit with high standard deviation amongst individual trees). The fact that oxo-phytodienoic acid (OPDA) may have a distinct signaling function, apart from being a precursor of jasmonate synthesis prompted us to specifically look for changes in the amount of this compound. Spruce accumulated OPDA only when the seedlings were in direct contact with the fungus (see Figure 3C).

The low-compatibility host pine, *P. sylvestris,* showed a different response. Abscisic acid did not respond to the fungus, while salicylic acid, OPDA and jasmonates increased with or without contact and irrespective of rosuvastatin repression of terpene synthesis (see Figure 3). This stimulation by the reduced volatilome indicates that additional non-terpenoid fungal volatiles are recognized by the tree.

In pine, oxo-phytodienoic acid responded similar to the jasmonates, compatible with its role as biosynthetic precursor. For jasmonates, individual compounds, free acids and conjugates were also investigated independently (Supplementary Table S1). Free jasmonic acid was the most prominent active compound in spruce in all treatments, and the levels of the isoleucine, valine and methionine conjugates varied between treatments (see Supplementary Table S1). Pine seedlings, however, produced jasmonates only in the presence of the fungus, irrespective of whether a direct contact between the partners was allowed for, or the presence of the full or reduced volatilomes. No active jasmonates, but only hydroxylated and carboxylated derivatives were detected in plants that were not exposed to T. vaccinum (see Supplementary Table S1). Jasmonic acid conjugates with phenylalanine or tryptophan were not detected. Thus, phytohormones on the whole did not respond specifically to the terpenes in the fungal volatiles.

### 3.3 Metabolome changes in response to fungal terpenoids

Since phytohormones showed a distinct, but not terpene-specific response, the downstream effects for plant metabolite production were assessed. Untargeted metabolomics were used and compounds showing significantly different abundances (*p* < 0.05; fold change >2) between the full and reduced volatilomes were further analyzed. Out of 970 different mass features detected in the compatible spruce trees, 20 compounds responded specifically to fungal terpenes with at least a two-fold change in abundance (Table 1). All were higher in abundance when the full volatilome was present.

**Table 1:** Metabolome of spruce (*Picea abies*) and pine (*Pinus sylvestris*) in treatments with the full or reduced volatilome of *Tricholoma vaccinum* in direct mycorrhizal contact or the tree only.

*Picea abies*														
			**Full volatilome**		**Reduced**		**Direct contact**		**Tree alone**		***p* f/r**	**-fold**	***p* f/c**	***p* f/t**
	RT	m/z	mean	SD	mean	SD	mean	SD	mean	SD				
Significantly different from control														
Monoterpene alcohol. C_10_H_21_O^+^ (Figure 4A)	15.28	157.159	32.78	18.36	2.66	4.61	0.00	0.00	15.44	2.29	0.01	12.31	0.09	0.01
Sesquiterpene oxide/alcohol. C_15_H_25_O^+^	11.67	221.190	52.56	30.01	9.50	6.22	6.46	6.52	34.97	17.96	0.02	5.53	0.38	0.02
Ornithine alkaloid. C_7_H_14_NO^+^ (Figure 4A)	1.17	128.107	105.33	54.79	17.53	30.36	27.15	33.37	0.00	0.00	0.02	6.01	0.01	0.03
Diterpenoid. C_20_H_31_^+^	18.12	271.242	162.12	75.42	44.14	33.14	53.83	55.08	12.29	8.72	0.02	3.67	0.01	0.03
Hydroxyflavonoid. C_15_H_13_O_6_	5.40	289.072	21.57	8.71	7.20	4.30	9.49	5.66	6.63	5.70	0.02	2.99	0.04	0.03
Phytosphingosine. C_18_H_40_NO_3_^+^ (Figure 4A)	16.09	318.301	122.31	70.19	41.49	9.43	52.65	21.48	37.14	16.63	0.049	2.95	0.04	0.08
Unknown. C_24_H_38_NaO_5_^+^	18.12	429.262	31.06	13.18	7.83	7.99	16.49	10.23	0.00	0.00	0.01	3.97	0.003	0.09
Diterpene acid. C_20_H_31_O_2_^+^	15.88	303.232	29.31	17.21	6.09	6.21	16.54	8.36	2.92	4.13	0.03	4.81	0.02	0.19
Unknown. C_18_H_14_NO_4_^+^	0.98	308.091	185.23	100.92	53.10	10.68	189.32	213.39	52.81	18.46	0.03	3.49	0.03	0.97
Non-significant versus control														
Unknown. C_10_H_17_O^+^	13.27	153.127	46.36	26.22	6.10	6.42	2.39	4.78	62.70	26.39	0.02	7.60	0.50	0.01
Diterpene acid. C_20_H_29_O_2_^+^	16.76	301.217	55.81	28.62	16.55	2.43	11.87	3.34	42.54	22.41	0.03	3.37	0.55	0.02
Diterpene alcohol/aldehyde. C_20_H_31_O^+^	19.49	287.237	111.54	54.45	31.90	17.05	25.27	4.26	86.44	54.20	0.02	3.50	0.61	0.02
Lignan. C_20_H_23_O_6_^+^	7.37	359.150	14.61	4.71	0.00	0.00	5.35	4.59	10.63	9.53	0.00	14.61	0.62	0.02
Diterpene alcohol. C_20_H_33_O^+^	16.36	289.253	234.09	160.18	27.83	11.48	29.89	17.92	180.58	119.74	0.03	8.41	0.65	0.04
Unknown. C_16_H_21_O_4_^+^	16.77	277.141	11.91	7.60	2.24	3.88	0.00	0.00	9.25	6.67	0.049	5.32	0.67	0.02
Unknown. C_12_H_19_O_2_^+^	15.73	195.137	33.57	17.77	12.72	4.69	6.54	9.61	29.09	14.63	0.049	2.64	0.75	0.02
Unknown	1.40	413.269	282.36	129.41	112.35	59.95	97.29	86.58	287.18	60.34	0.04	2.51	0.95	0.03
Flavonoid glycoside. C_21_H_21_O_10_^+^	6.94	433.114	327.42	172.08	114.00	61.43	122.48	49.14	332.07	96.32	0.04	2.87	0.97	0.04
Diterpene acid. C_20_H_29_O_2_^+^	21.22	301.217	79.29	31.74	32.08	8.75	20.01	14.05	57.08	28.80	0.02	2.47	0.42	0.01
Unknown. C_12_H_17_O_7_^+^	1.47	273.096	9.06	7.69	0.00	0.00	1.65	3.29	9.36	7.33	0.049	9.06	0.96	0.09
*Pinus sylvestris*														
Significantly different from control														
Diterpene acid. C_20_H_29_O_3_^+^ (Figure 4B)	16.34	317.211	222.00	70.60	97.63	26.36	97.42	31.59	49.41	6.02	0.02	2.27	0.01	0.02
Diterpene acid. C_20_H_27_O_2_^+^ (Figure 4B)	14.27	299.201	32.39	9.61	14.89	2.16	15.15	2.59	1.56	2.71	0.02	2.18	0.00	0.02
Diterpene acid. C_17_H_25_O_3_^+^	11.82	277.180	278.90	76.66	112.06	43.28	146.95	68.23	82.92	21.58	0.01	2.49	0.01	0.03
Diterpene acid. C_20_H_29_O_2_^+^ (Figure 4B)	15.09	301.217	26.99	9.35	11.42	2.55	13.10	3.90	3.36	3.38	0.03	2.36	0.00	0.04
Lower abundance with full volatilome; not significantly different versus control														
Sesquiterpene. C_15_H_25_^+^	20.59	205.196	30.83	25.45	68.21	11.33	60.57	25.22	20.45	16.32	0.04	2.21	0.53	0.14

RT, retention time; m/z. mass determination; SD, standard deviation; p, students *T*-test for significance treatments with full (f) and reduced (r) volatilome. Direct contact to Tricholoma (t) or tree without fungus as control (c); first columns indicate the compounds if identified; compatible tree (dark green) and the low-compatibility tree (light green) are given (*p* < 0.05; −fold difference > 2); last column red numbers indicate no statistically significant difference between full volatilome and direct contact with the tree; *n* ≥ 4.

**Figure 4:**
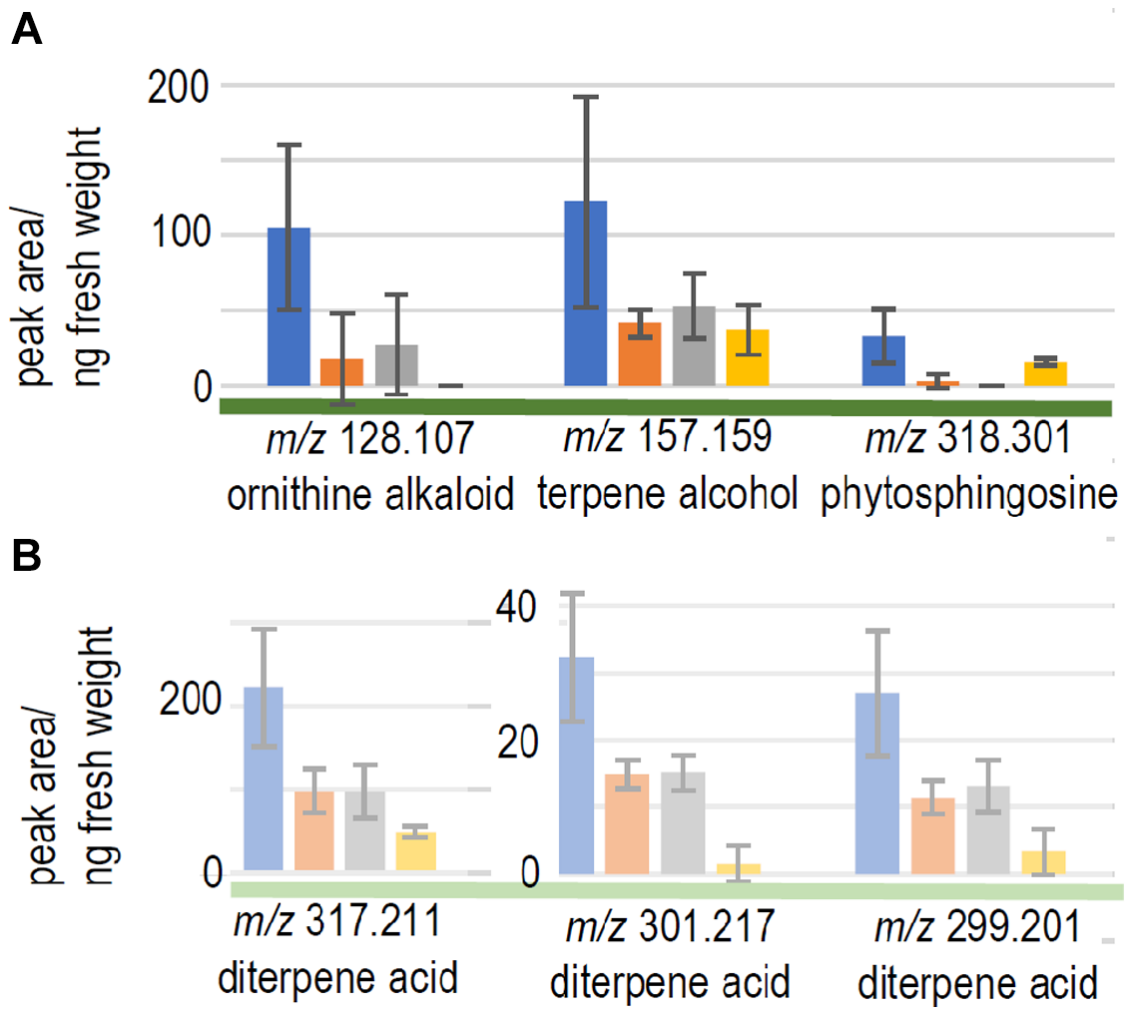
Metabolome changes of the host trees. **(A)** *Picea abies* or **(B)** pine were exposed to the full volatilome of *Tricholoma vaccinum* (blue), to the reduced volatilome (orange), in direct contact (grey) or in the control without the fungus (yellow), *n* ≥ 4.

We expected to see compounds, where already early contact via the exchange of volatiles led to changes in plant response. Therefore, the compounds with significant differences between control seedlings (without fungus) and the full volatilome exposure were further assessed. 9 compounds fulfilled all criteria (significant response to the full volatilome and significant change in response to the terpene-deficient volatilome), among them a putative ornithine alkaloid (*m/z* 128.107; Figure 4A). A monoterpene alcohol showed the highest fold-change, and another compound was identified as phytosphingosine (*m/z* 157.159 and *m/z* 318.301, respectively; Figure 4A). In addition to the terpenes, other compounds including flavonoids were detected. Five compounds in total were significantly different between full volatilome exposure and direct contact as well.

The response of the low-compatibility host was less pronounced and limited to terpenes (see Table 1). The metabolome of pine showed 771 compounds, of which only five showed significant difference between the responses to the full and reduced volatilomes, four with higher abundance in the full volatilome-exposed trees. The fold-changes in abundance were much less pronounced, the highest change being 2.5-fold. Among the interesting compounds responding to the full volatilome, three could be identified as diterpene acids, putatively lambertianic acid (*m/z* 317.211), dehydroabietic and abieta-tetraenoic acid (*m/z* 299.201 and 301.217, respectively). They were found to be significantly higher in extracts (*p* < 0.05) when terpenes from *T. vaccinum* were present in the headspace or direct contact was allowed. These metabolites were not detected when the fungus was absent (see Figure 4B). Thus, the metabolomes of the low-compatibility tree differed substantially from those in compatible interaction with the ectomycorrhizal fungus.

**Figure 5:**
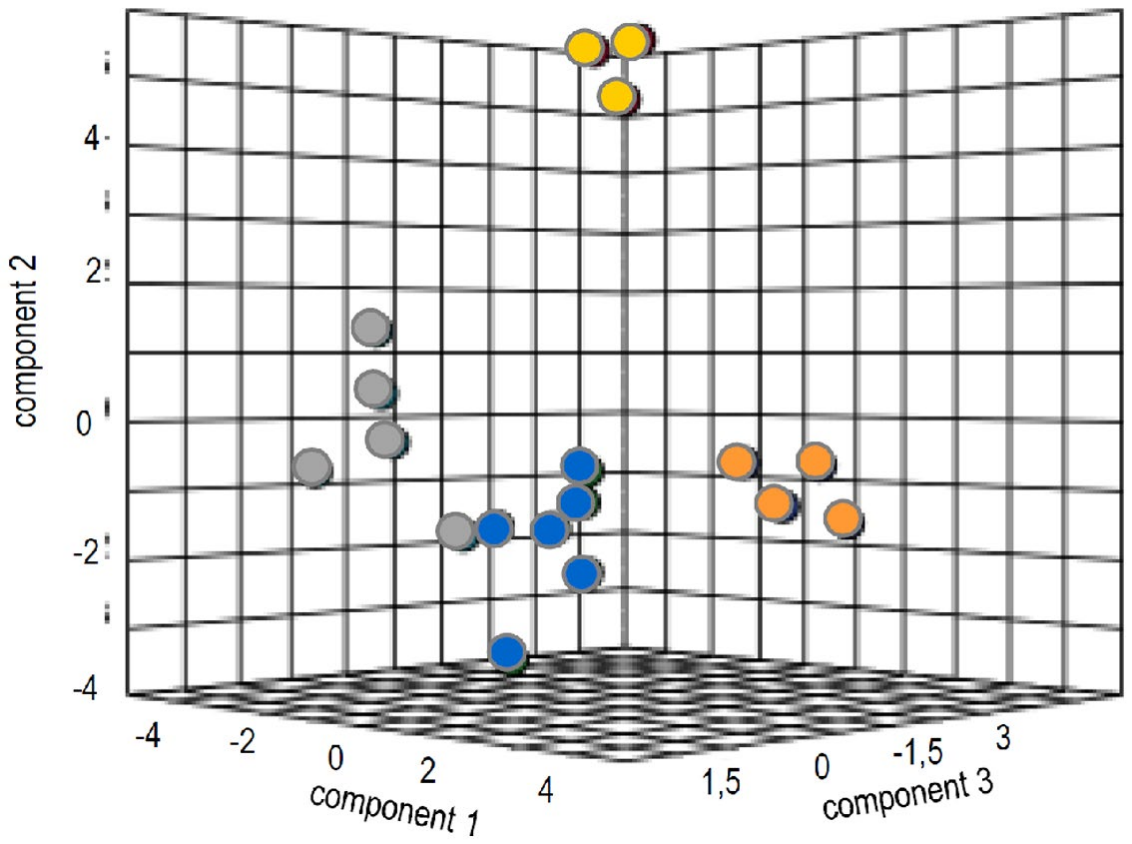
Sparse partial least square analysis of the metabolome and phytohormone changes in treatments with full volatilome (blue), reduced volatilome (orange), direct contact (grey) and without the fungus (yellow).

To identify general patterns of response to the ectomycorrhizal fungus, multivariate analysis was performed. The canonical correspondence analyses could verify tangible discrimination (35.2% for three components) between volatile-dependent and direct contact in the compatible host spruce (Figure 5). The evaluation for pine, as expected from the results, resulted in much less clear separation between treatments (Supplementary Figure S1).

## 4 Discussion

### 4.1 Smelling a friend at a distance

Both tree species showed distinct reactions to the presence of volatiles of the ectomycorrhizal fungus, *T. vaccinum*. As a part of that response, phytohormone levels changed. The compatible host spruce showed abscisic acid being specifically involved in the response to volatiles, while OPDA was seen in direct contact only. Abscisic acid is involved in plant responses to both biotic and abiotic stress, with a specific role in triggering secondary metabolism (Anderson et al., 2004). It has also been detected in response to mutualistic plant-microbe interactions including mycorrhization (Lievens et al., 2017). The observed 12-oxo-phytodienoic acid is known to be up-regulated by pathogen attack as well as by osmotic stress and wounding (Koch et al., 1999; Kessler and Baldwin, 2002). An interplay with OPDA has been described for abscisic acid leading to increased cell wall genesis and callus development (Dave et al., 2011). The dependency of phytodienoic acid on direct contact is in accordance with wounding being perceived only when the fungus is entering the root to establish the mycorrhizal tissue of a Hartig’ net.

For the low-compatibility host’s response, salicylic acid as well as jasmonates including the precursor OPDA were involved, again without terpenoids leading to differences in the answer. Salicylic acid is well known to mediate plant resistance against biotrophic attacks and is triggering systemic acquired resistance (Shah et al., 2014). In combination with abscisic acid, the response has been described for different, bacterial as well as fungal phytopathogens and thus is related to microbially-induced systemic acquired resistance described for roots of trees as well as for herbaceous plants (Pieterse et al., 2014). Jasmonic acid and its conjugates/derivatives are known to be induced by herbivory/wounding (Miersch et al., 2008; Pieterse et al., 2014). We could show that the trees clearly differentiated between recognition of a good versus mediocre root symbiont in early, volatile-based contact.

### 4.2 Terpenoids make a difference

The metabolic responses of the two tree species in question are different, as would be expected. We nevertheless could find indications of an ambiguous response in the low-compatibility host pine, while the response in the compatible spruce host was more pronounced and contained molecules that are more permissive for a root associate. Two putative abietic acid compounds were present specifically in response to fungal volatiles in the low-compatibility host, the diterpene acids dehydroabietic acid and abieta-8,11,13,15-tetraen-18-oic acid. These diterpenoids, as well as a third, lambertianic-like acid, were found to be enhanced in abundance by terpenes from *T. vaccinum*, while they were not detected (or present in minute concentrations) when fungal terpene synthesis was suppressed. Resin production would be a response upon stress activated in conifers, and abietic acid is the main constituent of amber (López-Goldar et al., 2020). Abietic acid has been shown to inhibit fungal spore germination and an abietane diterpenoid (dehydroabietinal) activated systemic resistance in *Arabidopsis thaliana* (Chaturvedi et al., 2012). This observation suggests that an induced systemic response may be mounted upon terpene recognition in the low-compatibility pine seedlings.

In the compatible host, spruce, more compound classes were elicited. The terpenes of the fungus elicited the formation of an ornithine alkaloid indicating a connection to the urea cycle, several mono-, sesqui-and diterpenoids, as well as phytosphingosine, a building block of sphingolipids. Diterpenes may interfere with gibberellic acid signaling (see Rosenkranz et al., 2021) and have been shown to possess antimicrobial activity, specifically against Gram-positive bacteria (Wilkens et al., 2002).

### 4.3 Modulators of plant immunity

Comparing the responses mounted to volatile terpenoids coming from the ectomycorrhizal *T. vaccinum* allowed us to identify different plant responses, although in the end, both lead to mutually beneficial symbioses. *T. vaccinum* shows high host specificity with a compatible and a low-compatibility host, that may explain these differential responses (Krause et al., 2015; Sammer et al., 2016). The phytohormone response showed that after terpenoid volatile signals indicated a root-associated microbe to *P. abies,* it mounted a microbially-induced systemic resistance. This response does not discriminate between pathogenic and symbiotic partners (Almario et al., 2022). In contrast, pine mounted a pathogen-related response. At the same time, this secondary host relied more on additional signals exchanged in full contact, indicating involvement of diffusible compound(s). The pine responded with induced systemic resistance leading to resin production hampering invasion, thus explaining the observed spotty and incomplete Hartig’ net formation in this low-compatibility interaction (Krause et al., 2015). In another ectomycorrhizal system, two fungal species altered tree physiology differently, again showing a specific interaction (Huang et al., 2023). There, two *Tuber* species were used on *Castanopsis rockii* seedlings and the authors could show lower organic carbon in the mycorrhizosphere, as well as higher tartrate and phosphatase for white Chinese truffle as compared to interaction with black Chinese truffle.

Using the interesting biological system shown here, with one fungus and two tree species, it cannot be excluded that the different responses are determined solely by the tree species. However, the evidence discussed above indicates that both trees respond specifically to a compatible or semi-compatible ectomycorrhizal fungus in accordance with root-associated responses in spruce and pathogen-associated signals in pine.

**Figure 6:**
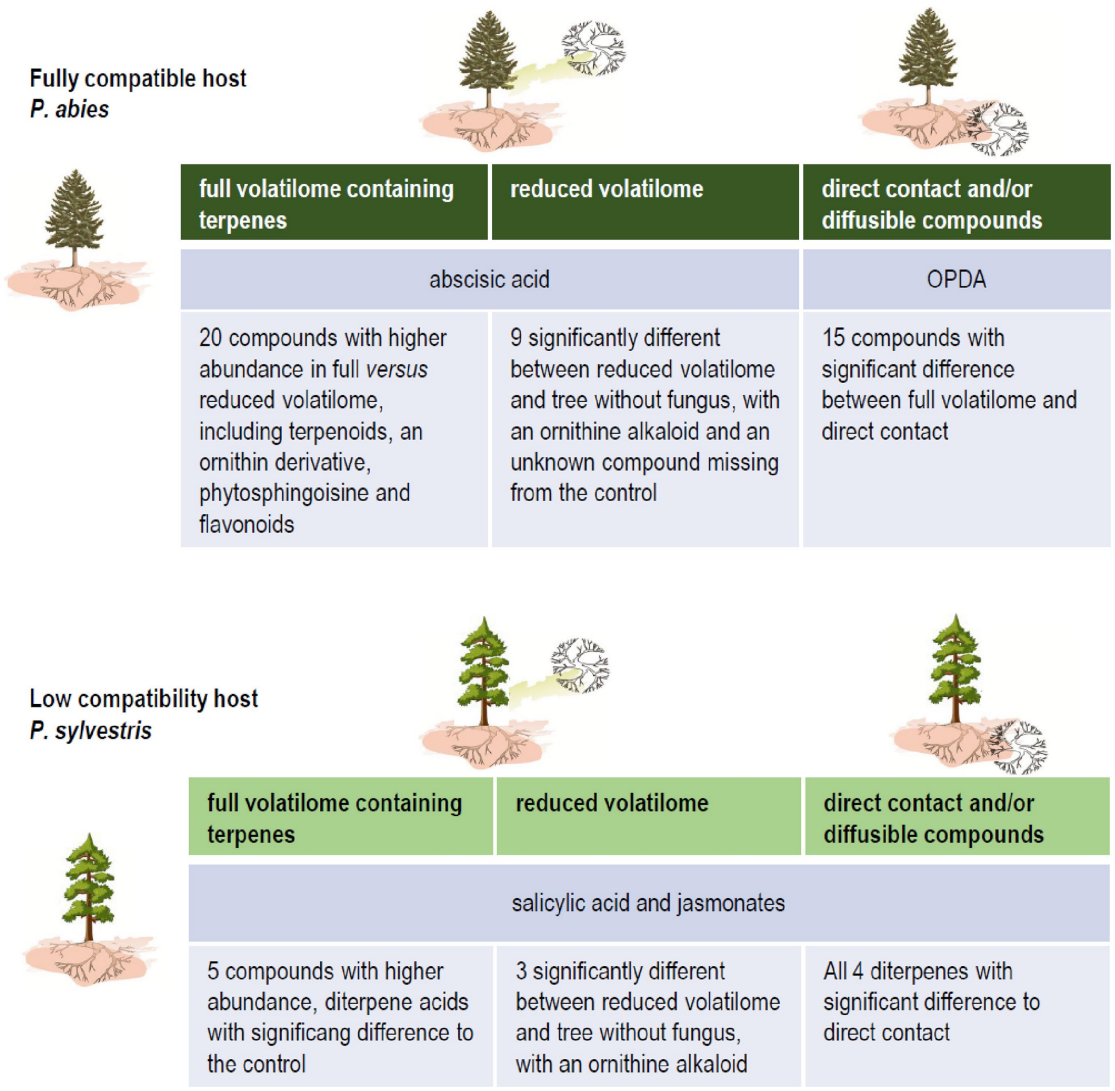
Impact of fungal terpenes on phytohormone levels and the metabolome of compatible and low-compatibility hosts spruce and pine, respectively.

The concepts for plant response originally have been developed in phytopathology. There, rather dichotomous decisions, like basal versus induced resistance, or local versus systemic responses were defined (compare Pieterse et al., 2014). A more flexible concept, in contrast, fits plant-microbe associations better. This confirms the concept of viewing interactions between pathogenic-neutral-mutually beneficial symbioses as a continuum based on constant signal exchange (Kothe and Turnau, 2018; Abdulsalam et al., 2019). With our comparison of compatible and low-compatibility ectomycorrhizae, we could support that view with new evidence indicating an earlier and more complete response with a cascade of reactions upon detection of terpenoids, full volatilome, and direct contact with the fungus (Figure 6). Nevertheless, the trans-kingdom interaction reacts to volatile exchange as an early, decisive factor in the interactions of ectomycorrhiza formation.

## Data availability statement

The datasets presented in this study can be found in online repositories. The names of the repository/repositories and accession number(s) can be found at: Bexis at the Data Management facilities of the Friedrich Schiller University Jena.

## Author contributions

ME: Investigation, Writing – original draft. RH: Methodology, Writing – review & editing. KK: Writing – review & editing, Conceptualization, Validation. WB: Formal analysis, Funding acquisition, Supervision. EK: Funding acquisition, Supervision, Writing – review & editing, Data curation, Project administration.
