# Supplementary material for: Pre-symbiotic response of the compatible host spruce and low-compatibility host pine to the ectomycorrhizal fungus Tricholoma vaccinum
Source: Front Microbiol. 2023 Dec 4;14:1280485. doi: 10.3389/fmicb.2023.1280485 (PMC10725908; doi:10.3389/fmicb.2023.1280485)
Supplement: Supplementary file 1 [file Data_Sheet_1.PDF]

Supplemental material

Suppl. Tab. S1: Individually quantified jasmonate signals, as free jasmonic acid and amino acid conjugates, after treatments with full volatilome (f), terpenes reduced by rosuvastatin treatment of the fungus (r), direct contact with tree allowed (t) or control without the fungus (c). Statistical significance for individual comparisons is given.

Picea abies											
[ng/g fresh weight]	full volatilome		reduced terpenes		direct contact		tree alone		p f/c	p r/c	p t/c
	mean	SD	mean	SD	mean	SD	mean	SD			
JA	2.31	2.76	2.13	1.78	1.96	1.49	0.62	0.94	0.25	0.25	0.18
JA-Ile	2.30	0.84	1.58	0.47	1.57	1	0.56	0.68	0.01	0.05	0.14
JA-Met	0	0	0.28	0.48	0.25	0.50	0.00	0	n.a*.	0.39	0.37
JA-Val	1.38	1.01	0.91	0.56	1.10	0.70	0.26	0.35	0.06	0.14	0.07
OH-JA	294.51	110.73	209.39	48.22	161.82	75.25	94.60	42.03	0.04	0.06	0.06
OH-JA-Ile	20.50	8.70	13.69	3.87	9.82	2.68	7.35	4.39	0.04	0.11	0.25
COOH-JA-Ile	3.65	3.50	1.97	1.19	1.17	0.67	0.90	1.16	0.08	0.14	0.94
Pinus sylvestris											
[ng/g fresh weight]	full volatilome		reduced terpenes		direct contact		tree alone		p f/c	p r/c	p t/c
	mean	SD	mean	SD	mean	SD	mean	SD			
JA	0.31	0.31	0.81	0.69	0.85	0.72	0	0	0.12	0.14	0.08
JA-Ile	0.36	0.37	0.41	0.26	0.58	0.29	0	0	0.12	0.07	0.02
JA-Met	1.14	0.57	0.66	0.50	0.31	0.26	0	0	0.02	0.11	0.08
JA-Val	0.36	0.38	0.48	0.14	0.33	0.26	0	0	0.13	0.01	0.06
OHJA	73.54	32.30	77.85	17.26	75.12	11.83	76.70	25.65	n.a.	n.a.	n.a.
OHJA-Ile	7.26	3.84	5.84	1.93	3.93	1.28	8.25	4.64	n.a.	n.a.	n.a.
COOHJA-Ile	0.46	0.93	1.17	0.89	0.60	0.53	0.00	0.00	n.a.	n.a.	n.a.

* n.a.. not applicable; mean. mean concentrations (n≥3); SD. standard deviation; OHJA, hydroxy-jasmonate; OHJA-Ile, hydroxy-jasmonoyl isoleucine; COOHJA-Ile, carboxy-jasmonoyl isoleuceine; full, head space with *T. vaccinum* terpenes; reduced, volatiles of *T. vaccinum* grown with the terpene synthase inhibitor rosuvastatin; contact, in direct contact with *T. vaccinum*; control, without the fungus.

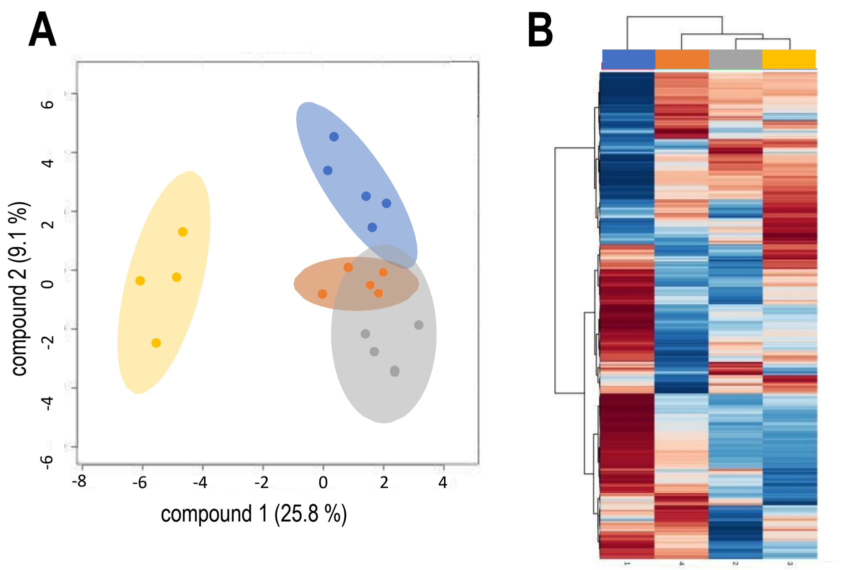

Suppl. Fig. S1: Overall dscrimivative impact of treatments on the metabolome of pine. (A) Partial least square discriminant analysis; (B) heatmap visualization of the metabolome analysis. The treatments with full volatilome (blue), reduced volatilome (orange), direct contact (grey) and control without the fungus (yellow) are given.
